# Changes In Actual And Perceived Physical Abilities In Clinically Obese Children: A 9-Month Multi-Component Intervention Study

**DOI:** 10.1371/journal.pone.0050782

**Published:** 2012-12-05

**Authors:** Milena Morano, Dario Colella, Irene Rutigliano, Pietro Fiore, Massimo Pettoello-Mantovani, Angelo Campanozzi

**Affiliations:** 1 Department of Biomedical Sciences, Motor Activities and Sport Sciences, University of Foggia, Foggia, Italy; 2 Department of Medical Sciences, Pediatrics, University of Foggia, Foggia, Italy; 3 Department of Medical Sciences, Physical Medicine and Rehabilitation Unit, University of Foggia, Foggia, Italy; University of Missouri-Kansas City, United States of America

## Abstract

**Objectives:**

(1) To examine relationships among changes in physical activity, physical fitness and some psychosocial determinants of activity behavior in a clinical sample of obese children involved in a multi-component program; (2) to investigate the causal relationship over time between physical activity and one of its strongest correlates (i.e. perceived physical ability).

**Methods:**

Self-reported physical activity and health-related fitness tests were administered before and after a 9-month intervention in 24 boys and 20 girls aged 8 to 11 years. Individuals’ perceptions of strength, speed and agility were assessed using the Perceived Physical Ability Scale, while body image was measured using Collins’ Child Figure Drawings.

**Results:**

Findings showed that body mass index, physical activity, performances on throwing and weight-bearing tasks, perceived physical ability and body image significantly improved after treatment among obese children. Gender differences were found in the correlational analyses, showing a link between actual and perceived physical abilities in boys, but not in girls. For the specific measurement interval of this study, perception of physical ability was an antecedent and not a potential consequence of physical activity.

**Conclusions:**

Results indicate that a multi-component activity program not based merely on a dose-effect approach enhances adherence of the participants and has the potential to increase the lifelong exercise skills of obese children. Rather than focusing entirely on diet and weight loss, findings support the inclusion of interventions directed toward improving perceived physical ability that is predictive of subsequent physical activity.

## Introduction

Although obesity prevalence seems to be stabilizing in several countries [1], child obesity is still considered a social concern because it tracks into adulthood and is related to adverse medical and psychosocial consequences [2]. Cross-sectional studies have found that obese children are more dissatisfied with their body image, perceive themselves as less physically competent, and perform poorly on both endurance and weight-bearing tasks compared with their normal-weight counterparts [3],[4]. Consequently, they tend to have a sedentary lifestyle and a negative attitude towards physical activity (PA) [5]. Findings have previously demonstrated that both actual and perceived physical competence (i.e. the individual’s perception of physical condition, sport and strength competence) are important correlates of PA and fitness in children and adolescents [6], but little research has investigated these relationships over time and, especially, in obese samples. A large body of evidence has attempted to validate the notion that PA increases body satisfaction through improvements in physical fitness and changes in perceived physical competence [7], but much of such research has been conducted on adults [8]. Furthermore, despite that weight loss and improving body appearance represent primary motivators for PA in obese children [9], evidence supporting the role of increased PA in childhood obesity management is weak [10].

Most interventions with a strong emphasis on increasing PA for the prevention and treatment of child obesity, have produced no or minimal effects on reducing obesity or improving PA participation [11],[12]. However, almost all existing studies have focused on changes in both diet and PA, making it difficult to assess the independent effects of these components and especially, the contribution of PA itself on the psychosocial aspects of childhood obesity [13],[14]. Evidence suggests that psychosocial benefits of increased PA in obese children are not always related to weight reduction, but it is still unclear whether improvements in psychosocial functioning may lead to increased PA in obese young people [15]. Data are therefore needed to determine whether engagement in PA is associated with changes in psychosocial determinants and whether these determinants influence PA behavior change in obese children. The evaluation of psychosocial correlates of PA as primary study outcomes, could provide strategies for promoting active behaviors, which in turn may lead to more effective interventions for treating child obesity.

To investigate PA behavior in children, researchers have adopted different theoretical approaches to exercise motivation, including the competence motivation theory [16] and the self-efficacy theory [17], which suggest that perceived competence is one of the most consistent predictors of PA. Children with high levels of actual and perceived physical competence may be more likely to engage in PA, because successful performance (i.e. increased actual competence) is expected to enhance perception of competence, which in turn affects motivation toward PA [18]. Perceived competence is therefore considered a mediator of the relationship between actual competence and PA [6], but its mediating role is an under-researched causal mechanism partially responsible for physical inactivity and thus for obesity [18]. Furthermore, research was unable to determine the direction of the PA-perceived competence relationship, and very little attention has been paid to the study of experimental or longitudinal associations between PA participation and the psychosocial determinants of behavior in obese children [19].

In order to develop and evaluate interventions aimed at increasing PA participation among obese children, there is a need to measure over time some factors that co-exist with obesity (e.g. low perceived ability, body image concerns, poor physical fitness), and to consider the interactions among them within a multidimensional approach. Therefore, in the context of a 9-month multi-component obesity program, incorporating fun-type skill-based physical activities, exercise training and a behavioral intervention, this study was designed: a) to analyze relationships among treatment-related outcomes – changes in body composition, physical fitness, and some psychosocial determinants of PA (i.e. perceived physical ability and body image), and also to examine gender differences; and b) to investigate the causal relationship over time between PA and one of its strongest correlates (i.e. perceived physical ability), using a two-wave two-variable panel design with a 9-month time lag. Specifically, our goal was to determine the temporal ordering of these two variables measured before (T_1_) and after (T_2_) the intervention program. Drawing on the causal priority approach [20], we hypothesized that positive reciprocal relationships would occur between physical activity levels (PAL) and perceived physical ability. It was expected that perception of physical ability at the start of the intervention would be directly related to and predict changes in PAL at T_2_ (hypothesis 1) and, vice versa, that PA at T_1_ would be positively associated with and precede changes in perceived physical ability over a 9-month period (hypothesis 2).

## Materials and Methods

### Participants

Forty-four obese children, 24 boys and 20 girls (mean age±SD: 9.2±1.3 years), were recruited through pediatricians at a city hospital in Puglia, one of the regions in southern Italy most affected by the prevalence of childhood obesity [21]. Participants who attended the outpatient clinic of the Centre for Pediatric Obesity at the University of Foggia, met the following inclusion criteria: (1) 8 to 11 years old and with a body mass index (BMI) equal to or greater than the 95^th^ percentile of the age- and gender-specific values on the Centers for Disease Control and Prevention (CDC) growth charts [22]; (2) without conditions that would limit PA, and (3) no regular participation in a structured exercise program outside the school.

### Ethics Statement

The study was conducted according to the Declaration of Helsinki and is part of a larger research protocol approved by the Institutional Review Board of the University of Foggia, Italy. All participants and their parents provided written informed consent prior to their enrolment in the study.

### The Physical Activity For Childhood Obesity (paco) Program

Based on the competence motivation theory [16] and the self-efficacy theory [17], the PACO program provided children with a wide range of fun-type skill learning opportunities in order to improve perceived and actual physical competence within a non-competitive environment. The overall goal for each child who attended the program was to develop a positive attitude toward, and a lifetime interest in, PA. The PACO program consisted of two or three 2-hour-long sessions per week (90 sessions delivered over 9 months), and was conducted by nine specialist instructors at the Physical Activity Centre of the local university. The intervention included educational content and practical experiences in the areas of health-related PA, exercise training and behavior modification. Drawing on PA and fitness recommendations for obese children [23], the content of each PACO lesson was based on activity or fitness components, including fundamental motor skills, aerobic fitness, strength, speed, and prevention of injuries via a variety of indoor and outdoor activities (i.e. mini and sport games, circuits, individual tasks). These activities were purposefully selected and adapted to enable participants to experience fun and success in their skill practice. Progression in PA demand was ensured by increasing the volume of activities (i.e. the number of exercises performed per session, the repetitions performed per set and the number of sets performed per exercise), and by modifying the weekly number of sessions from two to three at the mid-intervention point. Each session was mainly group-based, but participants also experienced a range of individual exercise modalities (i.e. walking, stepping, cycling, and seated rowing) for 25 minutes, with a child-to-instructor ratio of approximately 5∶1.

In addition to these supervised physical activities, participants were encouraged to be active outside the program and invited to keep an exercise diary at home. Behavioral skills training of children and families (i.e. goal-setting, self-monitoring, facilitative self-talk, self-reinforcement and recruiting social support) was provided in weekly 30-minute interactive group sessions. Each session included a review of the previous week’s goals, introduction of new topics, and goal-setting for the next week, in order to educate participants about the importance of fitness and increased PA. Training objectives also included teaching children about their bodies and promoting safe training procedures. As the primary goal of this study was to encourage the PA participation of each child by increasing his or her perceived and actual competences, weight loss *per se* was not explicitly discussed with participants. However, healthy eating habits were discussed with their parents and encouraged as part of the treatment, in the admission of children to the hospital outpatient clinic and with a repeated nutritional counselling at the midpoint of the intervention.

During the PACO program, the attendance was systematically recorded, including non-attendance due to exceptional circumstances (e.g. illness), and was scored as presence/absence (1 or 0) for each child. T_1_ and T_2_ assessments took place in four additional meetings a week before and a week after intervention.

### Measures

#### Body Composition

Anthropometric measurements were assessed by a single highly experienced pediatrician of the hospital, according to the recommendations of the International Society for the Advancement of Kinanthropometry [24]. Standing height to the nearest 0.1 cm was measured using a calibrated stadiometer (Seca 220, Hamburg, Germany), body weight was determined to the nearest 0.1 kg on a balance scale (Seca 761, Hamburg, Germany), and body circumferences (waist, hip, abdominal, and arm) were measured to the nearest 0.1 cm using a flexible nylon tape measure. BMI (kg/m^2^) was calculated from height and weight, and BMI centiles and *z*-scores were derived according to the CDC growth reference [22]. Skinfold thickness was determined to the nearest 0.1 mm at the biceps, triceps, subscapular and suprailiac sites using a Holtain skinfold caliper (Holtain Ltd, Crymych, UK). Triplicate readings were made at each site to improve the accuracy and the reproducibility of the measurements, and the mean of the three values was considered. The Brook’s equation [25] was used to predict total body density from skinfold thickness. The total body density was subsequently converted into the percentage of body fat via Siri’s equation [26].

#### Physical Activity

PA was assessed using the Physical Activity Questionnaire for Older Children (PAQ-C) [27], which is a self-administered 7-day recall instrument designed to measure moderate to vigorous PA in children ages 8 to 14 years old. The PAQ-C is a 9-item measure scored on a five-point scale anchored by 1 = little or no activity and 5 = very high levels of activity. Higher total scores indicate greater PA. Evidence was provided that supported the PAQ-C as a reliable and valid measure of general PAL in children grades 4 to 8 [27],[28].

#### Body Image

Body image was measured using Collins’ Child Figure Drawing [29], seven silhouette figures of boys and girls ranging from very thin to obese. The participants were asked to choose two pictures representing their current and ideal body shapes. Body dissatisfaction was then obtained as the self–ideal discrepancy, with positive and negative scores indicating a desire to be thinner and fatter, respectively. Moderate to high levels of test-retest reliability and validity data have been provided previously for the use of this scale with children [29].

#### Perceived Physical Ability

Individuals’ perceptions of strength, speed and agility were assessed using the Perceived Physical Ability Scale [30], which is composed of ten items structured in a response scale ranging from 1 =  Yes, very much to 5 =  No, not at all. For each item, children are required to choose the response that best represents their personal feelings. Items 1, 3, 5, 7, and 9 regard positive perceptions of physical ability (i.e. quick reaction and action, strength, motor control). The other five items assess the perception of the difficulty of movement (i.e. lack of control, clumsiness and muscle weakness) and are reverse-scored. Higher total scores correspond to a better perceived physical ability. Studies have supported the scale as a valid and reliable measure of perceived physical ability in children [3],[30].

#### Physical Fitness

Physical fitness was assessed using the following health-related fitness tests, which were reported to be reliable and valid methods in childhood and adolescence [31]–[33]:

- The standing long jump (SLJ) to assess lower-limb explosive strength. Participants were permitted to perform a countermovement with the arms and legs before jumping horizontally. The test was performed twice and was scored in centimeters. The longest distance jumped was reported.
- The medicine ball throw to evaluate upper-limb power. Children performed a two-hand overhead throw with a 1 kg medicine ball, from a standing position with feet slightly apart. The test was performed twice and was scored in meters. The highest value was used for statistical analyses.
- The 20-m sprint to assess speed and anaerobic power. Participants were instructed to cover the distance of 20 m as fast as possible. The test was scored in seconds.
- The 10×5-m agility shuttle run (ASR) to evaluate speed of movement, agility and coordination. Children were required to run back and forth five times along a 10 m distance at the highest speed possible. The time needed to complete the five cycles was scored in seconds.
- The 1-mile run/walk (1MRW) to assess aerobic power. Participants were instructed to cover the distance of 1 mile as quickly as possible. The time to complete the walk/run was scored in minutes. Heart rate was measured continuously throughout the test and recorded immediately after the walk/run using a Polar F4 Black Amber heart rate monitor (Kempele, Finland). Aerobic power (VO_2max_, ml/kg/min) was predicted from mile time, age, gender and BMI by using Cureton’s equation [34], which was developed on a large sample of children and adolescents.

### Statistical Analyses

A 2 (gender)×2 (time: T_1_ vs T_2_) analysis of variance with repeated measures was performed to examine the evolution of the the measured parameters in boys and girls over the two test periods. Effect sizes (ES) estimates were determined for all significant findings, with values of 0.2, 0.6, 1.2, and 2.0 indicating small, moderate, large, and very large ES, respectively [35].

To explore the relationships of perceived physical ability with physical fitness variables, Pearson’s correlation coefficients (r) were calculated for each time point (i.e. cross-sectional relationships) and for the change from baseline scores (i.e. longitudinal relationships). For each variable, the change score used in the correlational analyses, was the standardized residual obtained by regressing T_2_ score onto the respective T_1_ score. Unlike the alternative method involving differences (post- minus pre-scores), the residualized-change approach is recommended as it provides a change value, adjusted for baseline variance [36].

Multiple linear regression analyses using a cross-lagged procedure [20] were conducted to explore the causal ordering of PAL and perceived physical ability over a 9-month period. Gender (male = 1; female = 0) and age (in years) were included as covariates. To test whether perceived physical ability predicts changes in PAL (hypothesis 1), PA at T_2_ was first entered as the dependent variable, while the covariates and PA at T_1_ served as independent variables. Secondly, perceived physical ability at T_1_ was added. Then, the reverse causal hypothesis (hypothesis 2) was examined by regressing perceived physical ability at T_2_ on PA at T_1_ while controlling for gender, age, and the stability of the dependent variable at T_1_. Thus, for each of the two models, the effects of the covariates and the dependent variable at T_1_ (third variables) were controlled by including them in the regression analysis. This procedure allows for testing of the described diagonal paths or *crosslags* (i.e. each variable with the other at a different point in time), but also the longitudinal within construct paths (i.e. each variable with itself at two points in time). The cross-lagged-effects were of main interest, because they are usually regarded as the primary source of evidence for causal relationships between variables [37]. Reciprocity was supported if the cross-lagged associations were equal, and the direction was inferred if one relationship was significantly higher than the other. Statistical significance was set at p≤0.05. All analyses were conducted using SPSS version 17.0 (SPSS Inc., Chicago, IL, USA).

## Results

### Intervention Effects

All children completed the PACO program, and the mean overall number of sessions attended was 73.3±2.5 (81.4±2.7%), with an attendance range of 72 to 89 out of 90 sessions assigned. Descriptive statistics for each variable by gender and time of assessments are reported in Table 1. No gender × time interaction effects emerged, indicating that changes over time were similar for boys and girls. From T_1_ to T_2_, height (F_1,42_ = 93.8, p<0.001, ES = 0.5), weight (F_1,42_ = 9.0, p<0.004, ES = 0.2), and waist circumference (F_1,42_ = 4.5, p<0.04, ES = 0.1) significantly increased, whereas BMI (F_1,42_ = 8.8, p<0.005, ES = 0.2) decreased. Significant main effects of gender were found in the suprailiac skinfold thickness (F_1,42_ = 5.0, p = 0.031, ES = 0.6), BMI (F_1,42_ = 6.7, p = 0.013, ES = 0.8), arm (F_1,42_ = 5.3, p = 0.026, ES = 0.7) and waist (F_1,42_ = 9.3, p<0.004, ES = 0.9) circumferences, with boys reporting higher values than girls.

**Table 1:** Descriptive statistics by gender and time of assessments.

	Boys (n = 24)		Girls (n = 20)	
Variable	Pre	Post	Pre	Post
Height (cm)	138.1±10.7	141.9±10.4	137.6±7.5	142.5±7.1
Weight (kg)	52.1±13.5	53.6±13.4	46.3±7.4	48.5±6.8
BMI (kg/m^2^)	26.9±3.7	26.2±3.7	24.4±2.5	23.8±2.3
BMI *z*-score	2.3±0.4	2.2±0.4	2.0±0.3	1.9±0.3
BMI percentile	98.4±1.6	98.1±1.8	97.2±1.9	96.6±2.3
Body fat (%)	38.1±4.7	38.4±3.8	38.9±3.0	38.3±3.0
Skinfold thickness				
Biceps (mm)	19.4±7.8	19.2±6.0	17.4±4.1	16.5±3.9
Triceps (mm)	25.4±6.9	24.5±6.2	23.4±5.2	22.4±3.8
Subscapular (mm)	26.6±8.0	27.4±6.6	24.9±6.9	23.9±5.9
Suprailiac (mm)	26.1±7.4	26.9±7.2	22.3±6.0	22.6±5.7
Body circumferences				
Arm (cm)	27.2±2.9	27.1±3.2	25.1±2.0	25.6±2.3
Waist (cm)	78.9±9.0	80.3±8.8	72.6±5.7	73.4±4.5
Hip (cm)	87.5±11.3	90.1±10.9	87.0±5.9	88.2±5.2
Abdominal (cm)	87.7±10.7	87.8±10.6	83.4±6.0	84.3±6.0
Physical activity	2.1±0.5	2.5±0.7	2.1±0.4	2.5±0.5
Body dissatisfaction	2.0±1.3	1.6±1.0	1.9±1.0	1.3±0.6
Perceived physical ability				
Positive scale	17.4±3.7	18.3±3.5	16.8±2.5	17.4±2.8
Negative scale	19.5±3.7	21.3±3.6	20.7±3.1	22.5±2.5
Physical performance				
Standing long jump (cm)	86.8±20.0	97.0±19.9	97.7±10.3	108.8±11.6
Medicine ball throw (m)	3.9±1.0	4.5±1.0	3.7±1.1	3.9±1.1
20 m sprint (s)	5.3±0.8	5.1±0.8	4.9±0.4	4.9±0.3
10×5 m shuttle run (s)	28.0±3.8	26.7±2.9	25.6±2.2	25.0±2.0
One mile run/walk (min)	14.0±3.6	14.3±2.8	13.4±1.7	13.3±1.8
HR_max_ one mile (beats/min)	183.7±22.0	185.7±11.3	179.1±32.8	190.4±20.5
VO_2max_ (ml/kg/min)	49.9±10.6	49.9±5.1	38.6±3.0	38.3±2.5

Data are reported as mean ± SD; HR_ max_ = maximal heart rate; VO_2max_ = maximal oxygen consumption.

doi:10.1371/journal.pone.0050782.t001

For behavioral and psychological outcomes, significant time effects (p<0.001) were shown, with participants presenting lower body dissatisfaction (F_1,42_ = 12.2, ES = 0.5) and reporting higher PA (F_1,42_ = 15.9, ES = 0.6) and perceived physical ability (F_1,42_ = 18.3, ES = 0.5) scores after treatment compared with before entering the program. No gender differences were found in PA and psychosocial variables.

With regard to physical fitness, there were significant changes over time in the SLJ (F_1,42_ = 27.6, p<0.001, ES = 0.6), medicine ball throw (F_1,42_ = 6.1, p<0.02, ES = 0.4) and ASR (F_1,42_ = 12.7, p<0.001, ES = 0.3), with children showing better performances at T_2_ compared to T_1_. Significant main effects were consistently found for gender, with boys showing best performances in the SLJ and ASR (F_1,42_ = 6.2, p<0.02, ES = 0.7), and highest VO_2max_ (F_1,42_ = 44.2, p<0.001, ES = 2.0).

### Associations Between Real And Perceived Physical Abilities

Correlational analyses between actual and perceived physical abilities by gender and time of assessments were significant for boys, but not for girls. For male sample, at T_1_, perceived physical ability was unrelated to physical performance results, except for a positive relationship with the upper-limb power (r = 0.50, p<0.02) and a negative association with the 20-m sprint time (r = −0.52, p<0.01). At T_2_, correlational analyses indicated that perceived physical ability was positively related to the SLJ (r = 0.62, p<0.001), and negatively associated with the 1MRW (r = −0.59, p = 0.002), speed performances (20-m sprint: r = −0.64, p<0.001; ASR: r = −0.53, p<0.01) and VO_2max_ (r = −0.49, p = 0.01).

For boys, change from T_1_ in the perceived physical ability showed correlations with change from T_1_ in the 1MRW (r = −0.42, p<0.05) and with pre-treatment speed performances (20-m sprint: r = −0.44, p = 0.03; ASR: r = −0.51, p = 0.01). Finally, perception of physical ability at T_1_ was related to changes from T_1_ in the 1MRW (r = −0.51, p = 0.01) and VO_2max_ (r = −0.45, p<0.03).

### Across-Time Relationships Between Pa And Perceived Physical Ability

**Figure 1:**
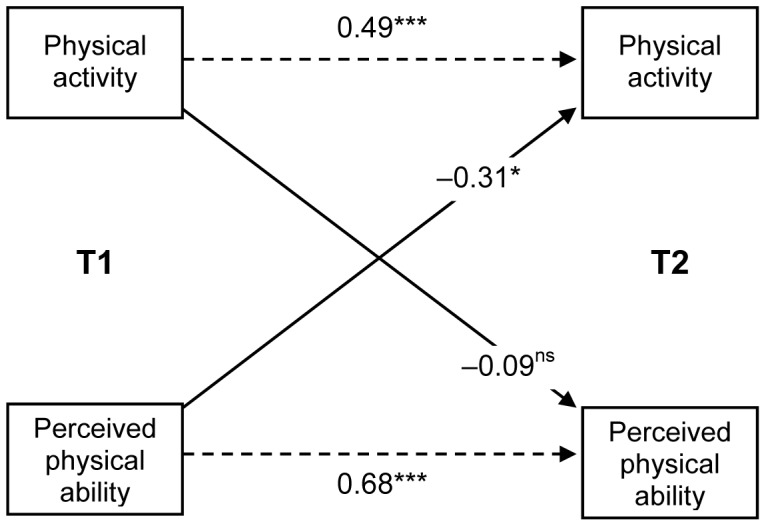
Across-time relationships between PA and perceived physical ability (n = 44). Standardized regression coefficients (β) are reported. Dashed arrows reflect the correlation of each variable with itself at two points in time (T_1_, T_2_); Solid arrows indicate the causal relationships over time between variables. *p = 0.02, ***p<0.001, ^ns^non-significant.

As shown in Table 2, baseline perceived physical ability was related to PAL at T_2_ (β = –0.31, p = 0.02) beyond PA at T_1_ (β = 0.49, p<0.001), and after controlling for participants’ gender and age. Children showing a lower perception of physical ability before the intervention, experienced the strongest increase in levels of PA nine months later. The second hypothesis predicted that the reverse relationship would also be significant. As the effect of PA at T_1_ on perceived physical ability at T_2_ was not significant (β = −0.09, *n.s.*), this hypothesis was not supported. Therefore, perception of physical ability and PAL were not reciprocally related across time (Figure 1).

**Table 2:** Multiple linear regression analyses testing for reciprocal relationships between physical activity and perceived physical ability (n = 44).

Dependent variables	EnteredVariables	β	F	R^2^
**Physical Activity (T_2_)**				
	Step 1		5.95***	0.31
	Gender	−0.02		
	Age	−0.20		
	PA (T_1_)	0.49***		
	Step 2		6.22***	0.39
	Gender	−0.07		
	Age	−0.13		
	PA (T_1_)	0.49***		
	PPA (T_1_)	−0.31*		
**Perceived physical ability** **(T_2_)**				
	Step 1		11.53***	0.46
	Gender	−0.07		
	Age	−0.09		
	PPA (T_1_)	0.68***		
	Step 2		8.72***	0.47
	Gender	−0.07		
	Age	−0.09		
	PPA (T_1_)	0.68***		
	PA (T_1_)	−0.09		

β = standardized regression coefficients; R^2^ =  square correlations values;

PA = physical ativity; PPA = percived physical ability; T_1_ =  time 1; T_2_ =  time 2.

* p = 0.02, ***p<0.001; unmarked β were non-significant.

doi:10.1371/journal.pone.0050782.t002

## Discussion

### Changes In Treatment-Related Outcomes

Obese boys and girls showed improvements over 9 months in BMI, PA, performances on throwing and weight-bearing tasks, perceived physical ability and body image. However, body satisfaction, perceived and actual physical abilities were lower than those reported in nonobese children of the same age and nationality [38]. No change was found in body composition and aerobic power of participants at the end of the program that, however, incorporated a multidimensional approach to increase PAL and not necessarily weight loss. To our knowledge, no longitudinal studies have been performed to evaluate simultaneously the independent effects of PA (i.e. not versus dietary modification) on physical fitness, perceived physical ability and body image in a similar clinically obese group. Although exercise and PA have been used in a range of childhood obesity treatments [12],[14],[39],[40], few studies, to date, have reported an increase in PA at post-test or follow-up [40].

Based on theories of motivation and behavioral change [16],[17], one under-researched approach to increasing PA participation among obese children is to enhance their self-perception and enjoyment by increasing their actual and perceived motor skill competence [18]. From this standpoint, it is not surprising that findings showed improvements in almost all study variables at T_2_. Within the PACO treatment, much attention was paid to the actual performance of PA and fitness in combination with experiences of success and body mastery, and the gradual increase in the volume of PA. Children performed a variety of activities tailor-made to enable them to increase actual and perceived physical competences which, in turn, might help participants to be less conscious of body weight and thus more likely to become physically active. Martin and Lichtenberger [7] have argued that improvements in perceived physical abilities could reduce an individual’s body dissatisfaction through programs aimed at improving body mastery rather than physical appearance. Body concerns are often considered a barrier to PA, because programs focusing directly on body image may inadvertently heighten weight consciousness among obese children, who already consider self-image problematic prior to any intervention [41]. From this viewpoint, our findings could have practical implications for educators and trainers in identifying focus areas when designing child obesity programs.

In contrast with many other approaches which have used fixed intensities, duration and frequency of prescribed exercise sessions [39], in our intervention the only change is in the volume of PA. As the major challenge of the PACO program was motivating obese children towards PA, we considered the gradual change of volume most likely to provide opportunities for participants to experience a sense of accomplishment during their skill practice. Although this study was uncontrolled, the results support a previous randomized trial showing that a program modified by increasing the volume of PA among obese children can enhance perceived physical competence and body satisfaction independently of changes in body composition [15]. The Authors [15] found that overall volume of PA was more strongly related to self-perception improvements than intensity, probably because sustaining bouts of high intensity PA may affect adherence to the program, resulting in reduced psychosocial health benefits.

The PACO program provided children with intermittent, task-oriented activities, which however, may not trigger aerobic responses. The use of such a strategy could therefore explain why no change over time was found in the body composition, 1MRW performance and VO_2max_ of participants. Although PA of moderate-to-high intensity should be expected to improve body composition and VO_2max_ [14],[39], research found an adverse secular change in cardiorespiratory fitness and overweight among prepubertal children [42]. As suggested by Rowland [43], the diminished level of physiologic aerobic trainability in children could be related to the beginning VO_2max_ value, which was already high in this study sample. According to the Healthy Fitness Zone Standards for VO_2max_ (range 42–52 and 39–47 ml/kg/min for 10-yr.-old normal-weight boys and girls, respectively) [31], boys in the current sample may seem to be as aerobically fit, but obese individuals require greater aerobic power to move their heavier body mass. Therefore, the true association between body composition and VO_2max_ is more complex because of the relationship both variables have with PA. While physical training is usually associated with favorable changes in body composition [14], there is no strong relationship between PAL and VO_2max_ in the pediatric population [43]. It seems thus probable that the lack of developmental improvement in aerobic power of our study participants is related not only to the time devoted to this capability during the program, but also to some factors (i.e. body composition, prepubertal status, running/walking efficiency and economy, pre-training VO_2max_ value, motivation during testing experience) which might influence the aerobic trainability of obese children. Further research on the contrasting relationships of PA with aerobic fitness and body fatness among boys and girls is required.

### Gender Differences In Intervention Response

Next to the differences between boys and girls in the VO_2max_, the influence of gender was observed on the magnitude of changes in BMI and in some physical performances (i.e. SLJ and ASR). In addition to poorer levels of physical fitness, girls are generally less active and report lower perceived physical ability and greater body dissatisfaction than boys in a school setting [3],[38],[44],[45]. Against expectations, our findings showed no gender differences in these behavioral and psychological outcomes at T_1_ and T_2_, suggesting that perception of physical abilities and body image may not vary between boys and girls within an environmental context in which all children are obese, involved in a structured PA program and placed in noncomparative (or noncompetitive) situations.

Gender differences were found in the correlational analyses, showing a link between actual and perceived physical abilities in boys, but not in girls. Therefore, enhancing physical fitness in obese girls would not influence their perception of ability, as much as could happen in boys. Males and females perceive physical activities in different ways, and thus perceived physical ability may vary depending on the meaning they ascribe to those activities, and may not necessarily be related to actual physical performance, because of varying levels of comfort with their body or unrealistic and idealistic notions of performance.

For boys, correlational analyses showed that perceived physical ability was associated with aerobic fitness for most of the relationships explored. However correlations between perception of physical ability with the 1MRW and VO_2max_ were stronger at T_2_ compared to change from T_1_ scores, suggesting that changes in perceived physical ability and changes in aerobic performances track differently over time. Correlation coefficients also showed that baseline perception of physical ability was correlated with changes in aerobic fitness, but it was unrelated to changes in the other physical performance results. These findings indicate that there is no relationship between perceived physical ability at T_1_ and treatment-related changes in tasks requiring horizontal propulsion and vertical lifting of the body mass. Thus, it could be suggested that obese boys may achieve increased physical performances in weight-bearing tasks with intervention, irrespective of their level of baseline perceived physical ability, which, on the other hand, may influence improvements in aerobic fitness. Finally, it was found that pre-treatment speed times were negatively related to change in the perceived physical ability, which therefore may be directly influenced by the baseline level of these performances. Based on the present findings, the type of exercise used, especially in the early stages of a program for inactive children, appears to be important in bolstering the participants’ perception of physical ability and, consequently in helping them establish PA patterns.

### Perceived Physical Ability As Predictor Of Changes In Pa

Surprisingly findings revealed that PA and perceived physical ability do not have reciprocal causal relationships across time. Indeed, in the current sample and for a 9-month measurement interval, perception of physical ability was merely an antecedent to, and not a potential consequence of, PA. Over time, perceived physical ability became an increasingly important predictor of change in PAL especially in children who perceived themselves to be less physically competent at T_1_. In the context of the PACO intervention, which was aimed at improving perceived physical ability, it is interesting to note that children with the lowest baseline ratings of perceived physical ability were the ones experiencing the largest increase in PA over the 9-month period. Their perceived physical ability (and consequently their PA) had increased more in response to the intervention, compared to children with already high perceived physical ability at T_1_. This result may suggest that the PACO program was effective in improving perceived physical ability, given that children with low perceived physical ability at T_1_ have benefited more from such a program.

Previous studies have already found that perceived physical competence is an important correlate of PA [44],[45], but unlike these cross-sectional investigations, we have captured the causal relationship between variables while the study was ongoing. Despite the fact that the results of cross-lagged panel analysis cannot be generalized beyond the specific measurement interval used in this study, findings could have methodological implications by suggesting that the first step in encouraging obese children to exercise could be to increase their perception of physical ability, which may lead to, and not result from, a more sustained participation in PA.

### Strengths And Limitations

A key strength of the PACO intervention was its acceptability to participants. The mean attendance was high and all children who started, completed the program, indicating that it was well-tolerated and suited the needs of the participants. Other strengths of our study included: (1) the adoption of a multi-disciplinary approach to examine the evolution of PA, physical fitness and some psychosocial determinants of activity behavior; (2) the longitudinal study design which allowed us to address the issue of directionality between PA and perceived physical ability in children treated for obesity. However our findings should be interpreted with caution, as they are based on a restricted size and selective sample, and it should be noted that the data on PA and perceived ability were obtained by self-reports. Even though they could have been affected by social desirability, we were careful to use valid and reliable instruments, and the Perceived Physical Ability Scale [30] was also previously tested in a population of obese children [3]. Finally, and more importantly, the study is limited because we utilized a one-group, pre- and post-test study design. As such, results could not be compared with data from a control group and should be considered preliminary, despite providing interesting findings for follow-up research. Long-term follow-up studies with multiple evaluations are needed to better investigate the relationships among physical, psychological and behavioral factors, and to determine whether changes in these treatment-related outcomes are maintained after the intervention is stopped.

### Conclusions

Results indicate that a multi-component activity program not based merely on a dose-effect approach enhances adherence of the participants and has the potential to increase the lifelong exercise skills of obese children. This is promising, especially considering that exercise drop-out has been highlighted as one of the major causes that render exercise prescription useless. In contrast to interventions focusing on diet, changing PA is less directly linked to a prohibition of activities and may thus be perceived by participants as positive rather than negative. When working with obese children who typically have limited experience participating in a structured exercise program, as well as low physical self-perception, it seems reasonable to suggest that educators and trainers should provide a variety of sustainable activities in which all participants can feel good about their bodies and their performance, and get excited about monitoring their progress. Rather than focusing entirely on weight loss, findings support the inclusion of interventions directed toward improving perceived physical ability that is predictive of subsequent PA.
